# Spatial variation in lymphatic filariasis risk factors of hotspot zones in Ghana

**DOI:** 10.1186/s12889-021-10234-9

**Published:** 2021-01-28

**Authors:** Efiba Vidda Senkyire Kwarteng, Samuel Ato Andam-Akorful, Alexander Kwarteng, Da-Costa Boakye Asare, Jonathan Arthur Quaye-Ballard, Frank Badu Osei, Alfred Allan Duker

**Affiliations:** 1 Department of Geomatic Engineering, Kwame Nkrumah University of Science and Technology, Kumasi, Ghana; 2 Department of Biochemistry and Biotechnology, Kwame Nkrumah University of Science and Technology, Kumasi, Ghana; 3 Department of Earth Observation Science, University of Twente, Enschede, Netherlands

**Keywords:** Lymphatic filariasis, Machine learning, Ensemble modelling, Generalised boosted model (GBM), Random forest (RF), Ecological niche modelling

## Abstract

**Background:**

Lymphatic Filariasis (LF), a parasitic nematode infection, poses a huge economic burden to affected countries. LF endemicity is localized and its prevalence is spatially heterogeneous. In Ghana, there exists differences in LF prevalence and multiplicity of symptoms in the country’s northern and southern parts. Species distribution models (SDMs) have been utilized to explore the suite of risk factors that influence the transmission of LF in these geographically distinct regions.

**Methods:**

Presence-absence records of microfilaria (*mf*) cases were stratified into northern and southern zones and used to run SDMs, while climate, socioeconomic, and land cover variables provided explanatory information. Generalized Linear Model (GLM), Generalized Boosted Model (GBM), Artificial Neural Network (ANN), Surface Range Envelope (SRE), Multivariate Adaptive Regression Splines (MARS), and Random Forests (RF) algorithms were run for both study zones and also for the entire country for comparison.

**Results:**

Best model quality was obtained with RF and GBM algorithms with the highest Area under the Curve (AUC) of 0.98 and 0.95, respectively. The models predicted high suitable environments for LF transmission in the short grass savanna (northern) and coastal (southern) areas of Ghana. Mainly, land cover and socioeconomic variables such as proximity to inland water bodies and population density uniquely influenced LF transmission in the south. At the same time, poor housing was a distinctive risk factor in the north. Precipitation, temperature, slope, and poverty were common risk factors but with subtle variations in response values, which were confirmed by the countrywide model.

**Conclusions:**

This study has demonstrated that different variable combinations influence the occurrence of lymphatic filariasis in northern and southern Ghana. Thus, an understanding of the geographic distinctness in risk factors is required to inform on the development of area-specific transmission control systems towards LF elimination in Ghana and internationally.

## Background

Lymphatic filariasis (LF) is one of the neglected tropical diseases (NTDs), which presents chronic disabling and disfiguring pathologies with occasional painful attacks on affected persons [35]. LF is a mosquito-borne infection caused by filarial nematodes: *Wuchereria bancrofti*, *Brugia timori,* and *B. malayi* [34]. These worms produce larvae i.e., microfilariae (*mf*) - transmitted by mosquitoes in endemic areas; thus, reducing *mf* levels is significant towards LF eradication [18]. It is estimated that over 1.4 million individuals are at risk of infection in 83 endemic countries [7]. Currently, the mainstay eradication strategies, which include Mass Drug Administration (MDA) and vector control, have significantly interrupted LF transmission in many previously endemic settings [13]. While these achievements are commendable, there is the need to adopt novel approaches, especially in foci, where LF transmission is ongoing despite several years of implementing these control strategies.

In Ghana, studies on LF have shown differences in disease prevalence and multiplicity of symptoms in two geographically distinct regions, i.e., the northern and southern parts [23]. The northern regions of the country exhibit higher prevalence compared to the southern regions, but the middle forest belt is relatively free from the infection ([23]: [14]). Elsewhere, a study has revealed some level of genetic variability in parasite strains in the two endemic areas [9]. Furthermore, Pi-Bansa et al. [32] identified a vector of very high vectorial capacity specific to the coastal areas i.e., southern Ghana. These variations in the two regions could be due to different climatological, land cover, and socioeconomic risk factors.

De Souza et al. [10] reported that ecological and climatic variables such as elevations greater than 200 m, mean daily precipitation between 2.6–3.8 mm, and mean daily temperature range between 24.5–26.0 °C, influence the distribution of *Anopheles gambiae,* one of the vectors for LF transmission in Ghana. At the global scale, another study used climatic and environmental variables in a boosted regression tree (BRT) model to map the transmission limits of LF [6], confirming the influence of geo-environmental risk factors on vector population and vectorial capacity [17, 11].

While these studies present very useful findings, their spatial scale of analysis obscures some micro-level risk factors [28], which may be important for designing disease control strategies, especially in hotspots zones. According to Williams et al. [40], the spatial scale for analysis should include the known environmental or geographic limits of the species under study for quality model predictions. In the West African sub-region, different geographical zones have been documented [11]. The south is characterized by wetlands, while the north is characterized by drylands and sub-Sahelian climate [32]. In Ghana, the northern and the southern regions, although both highly endemic for *W. bancrofti* infections have distinct geographic characteristics (i.e., land cover and climate). This distinction is likely to influence vector proliferation and transmission potential differently.

Therefore, to facilitate LF elimination in these two highly endemic areas, a local understanding of the environmental, climatic, and socioeconomic factors that drive transmission is required to review existing control programmes. In line with this, the present study sought to map the environmental niches of LF and examine the behaviour of the diverse risk factors that drive transmission in Ghana’s northern and southern zones. Since the prevalence of LF is denoted by the presence or absence of microfilaria (*mf*) cases, data on *mf* survey from sentinel and spot-check sites across Ghana were stratified into the northern zone (NZ) and southern zone (SZ). These were used to run Species Distribution Models (SDMs), while climate, socioeconomic and land cover variables were used as covariates. The analysis was then performed over the entire country (countrywide (CW)) for comparison.

The remainder of the manuscripts is organized as follows. First, we evaluated and selected the range of risk variables influencing the occurrence of LF in the study zones. Second, six SDMs were selected for the mapping of environments suitable for LF transmission. Third, we described and compared the response curves of observed covariates on the probability of LF occurrence in the NZ and SZ study zones.

## Methods

### Study area

**Fig. 1:**
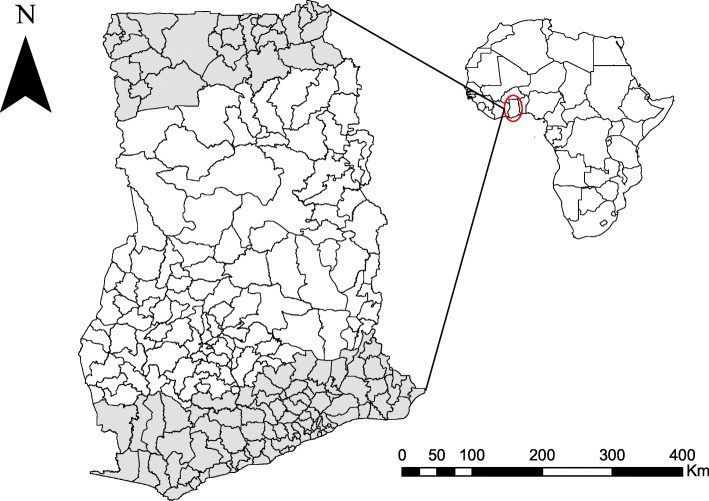
Map of Ghana showing the districts included in the two study zones, NZ and SZ shaded in grey. (This map was generated by authors with ArcGIS V.10.6 software (ESRI, Redlands, CA, USA) and no permissions are required to publish it)

The study was conducted in Ghana, as shown in Fig. 1. However, because LF appears to be localized in northern and southern Ghana, the study area was subdivided only to include highly endemic areas in these two zones. To investigate risk factors in the two highly endemic zones and how they compare with the result from the entire country, three zonal analyses were performed: countrywide (CW), NZ, and SZ. The area considered as the SZ in this study included districts that lie along the coastal savannah, tropical rainforest and some portion of Ghana’s moist semi-deciduous forest region, while the NZ comprised the Sudan savannah and some part of the Guinea savannah.

The NZ lies in the dry Guinea Savannah Ecological zone [32] with a sub-Sahelian climate made up of a wet and a dry season. The wet season extends from April to October, with a mean annual rainfall of approximately 1365 mm. Similarly, the dry season is subdivided into the Harmattan from November to mid-February and the dry, hot season from mid-February to April. Monthly temperatures range from 20 °C to 40 °C.

In contrast, the SZ lies within the high rain forest ecological zone of the West African sub-region, with strands of mangroves [11] and lots of wetlands. The climate in this region is tropical, characterized by two distinctive seasonal rainfalls; a major one between April and June and a minor one that occurs between September and October. The relative humidity is generally high, averaging between 75 to 85% in the rainy and 70 to 80% in the dry seasons. The highest mean temperature is 34 °C, whereas the lowest is 20 °C.

### LF prevalence data

**Fig. 2:**
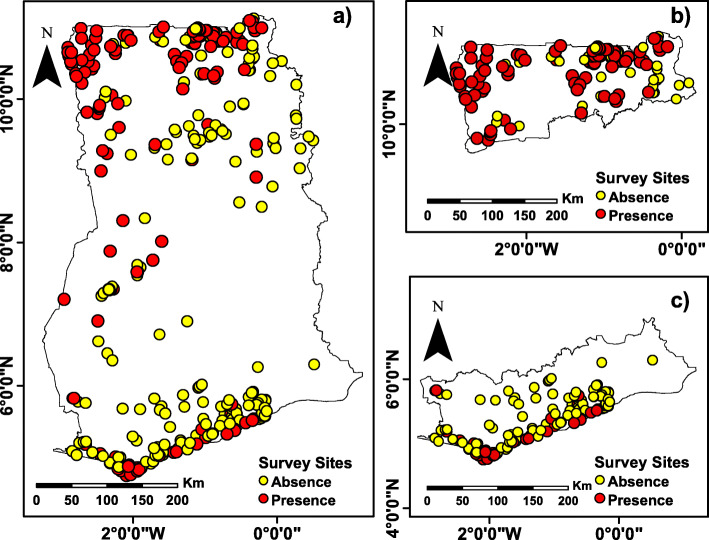
*mf* cases for surveyed communities from 2000 to 2014 (yellow indicates absence and red indicates presence), a) CW, b) NZ and c) SZ Zones. (This map was generated by authors with ArcGIS V.10.6 software (ESRI, Redlands, CA, USA) and no permissions are required to publish it)

Data on *mf* cases in Ghana was obtained from published articles in peer-reviewed journals ([2]: [22]). The data spanning 2000 to 2014 contained information on the year samples were collected, the number of years of MDA, the number of people examined, and the number of *mf* positive cases recorded for each study community. In all, 430 communities were surveyed for LF infections as part of a transmission assessment survey in Ghana. Details of this dataset were described by Biritwum et al. [2]. Spatial locations of these communities were extracted from multiple sources, including Google Earth Pro, Open Street Map, directory of cities and towns (world database), and database of the Ghana National Identification Authority card registration projects. Figure 2 shows a map of the spatial distribution of *mf* cases in Ghana (Fig. 2a), the NZ (Fig. 2b), and SZ (Fig. 2c).

### Geo-environmental and climatological data source

To identify the combination of explanatory variables that create a suitable environment for the transmission of lymphatic filariasis, land cover, socioeconomic and climatic predictors were obtained from various remotely-sensed datasets. Enhanced Vegetation Index (EVI) was generated from the Moderate Resolution Imaging Spectro-radiometer (MODIS) satellite image, specifically MOD13Q1 v006 [30]. This data is generated every 16 days at 250 m spatial resolution.

From the United States Geological Surveys (USGS) earth explorer project (*US* [39]), a raster dataset of elevation produced by the Shuttle Radar Topography Mission (SRTM) and Slope covariate were derived. Additionally, Landsat 7 ETM + 1 level 1 at 30 × 30 m resolution of less than 1% cloud cover was downloaded from the same site for Land Use/Land Cover (LULC) classification.

To determine rural and mostly poor areas in Ghana, Night-light emissivity from 2000 to 2014 captured by the operational linescan system instrument was used as a proxy [16]. This instrument measures visible and infrared radiation emitted at night time. The values range from 0 to 62, representing undetectable emissivity and maximum emissivity, respectively. Night-light emissivity has been shown to correlate with economic development in subnational regions of developing countries [5]. Another socioeconomic variable used was housing prevalence with improved drinking water and sanitation, sufficient living area, and durable construction across sub-Saharan Africa [38]. The prevalence of houses built with finished materials is higher in urban areas than in rural areas showing 84 and 34% improvement, respectively.

Precipitation and temperature variables were downloaded from the WorldClim database [41]. This dataset provides a set of global climate layers obtained by interpolation of weather station datasets distributed across the world. Other covariates used in the SDMs with details on the sources are provided in Table 1. Input grids were resampled to a common spatial resolution of 1 km^2^ using bilinear resampling for analysis performed with CW data. In contrast, a finer resolution of 250 m^2^ was used for the NZ and SZ to capture detailed information [40]. Raster layers were coerced to the same boundary extent to enable stacking for analysis. Raster manipulation and processing were undertaken using raster package in R V.3.5.3 and final map layouts created with ArcGIS V.10.6 software (ESRI, Redlands, CA, USA).

**Table 1:** Environmental variables used in the SDMs for mf occurrence and their sources

Variable	Variable Description	Source
Population	Population Density	WorldPop [26]
Housing	Improved Housing	The malaria atlas project [38]
DEM	Digital elevation model	STRM (US [39])
Waterbodies	Proximity to all water bodies and wetlands; swamps and marshes	
Slope	Derived from elevation	
LULC	Land use and land cover classes	Landsat 7 (US [39])
Bio 1	Annual Mean Temperature	WorldClim [41]
Bio 12	Annual Precipitation	
Bio17	Precipitation of the direst quarter	
Bio 18	Precipitation of Warmest Quarter	
Bio 19	Precipitation of Coldest Quarter	
NTL	Distance to stable night light	
MeanDayLST	Mean Day Land Surface Temperature	MOD11A2 [30]
MaxDayLST	Maximum Day Land Surface Temperature	
MinDayLST	Minimum Day Land Surface Temperature	
MeanNightLST	Mean Night Land Surface Temperature	
MaxNightLST	Maximum Night Land Surface Temperature	
MinNightLST	Minimum Night Land Surface Temperature	
EVI	Enhanced Vegetation Index	MOD13Q1 [30]

### Variable selection and model development

To identify the optimal suite of covariates to include in the specie distribution models, the variables were grouped into three categories; land cover, socioeconomic and climatic variables [29]. A test for variable collinearity with the Variance Inflation Factor (VIF) diagnostic method was adopted within each group. Since there are no formal criteria for deciding when a VIF is too large, a generic cutoff value of *VIF* ≥ 10 was used [8]. This approach reduces any potential collinearity and confounding effects such that for *p* - 1 independent variables,

1 $$ {VIF}_i=\frac{1}{\ 1-{r}_i^2},i=1,\dots \dots \dots \dots, p-1, $$

where ${r}_i^2$ is the coefficient of determination obtained by fitting a regression model for the *i*th independent variable on the other *p* − 2 independent variables. After the collinearity check, only Bio1 (Mean Annual Temperature) had a collinearity problem.

### Variable relative contribution

After strongly correlated variables were removed, the range of variables influencing the occurrence of *mf*, were identified using boosted regression trees (BRT). This method draws insights and techniques from both statistical and machine learning traditions. The advantage of this method over the others is its strong predictive performance and consistent identification of relevant variables and interactions. Here, the probability of *mf* occurrence, *y* = 1, in a sampled community with covariates X, is given as *p*(*y* = 1| *X*). This probability models via a logit function *f*(*x*) = *p*(*y* = 1| *x*).

Analytically, BRT regularization involves jointly optimizing the number of trees (*nt*), learning rate (*lr*), and tree complexity (*tc*). The optimal number of trees was estimated by the default 10-fold cross-validation (CV) method [15]. With a slow enough *lr of* 0.01, the CV estimates of *nt* are reliable and close to those from independent data. To ensure the modelling of possible interactions between predictors, a *tc* of 5 was selected. A *tc* of 1 fits an additive model, while a *tc* of 2 fits a model with up to two-way interactions, and so on [15]. It has been proven that stochasticity improves model performance, and fractions in the range of 0·5–0·75 have given best results for presence–absence responses [15]. Therefore, a bag fraction of 0·75 and an error structure of Bernoulli was used from here on.

The relative importance of the variables was computed by measuring the number of times a predictor variable is selected for splitting, and weighted by the squared improvement to the model as a result of each split, then an average over all the trees is determined [20]. Expressing in mathematical terms, the relative influence, ${\hat{I}}_j$ of the input variables *x*_*j*_ for a collection of decision trees ${\left\{{T}_m\right\}}_1^M$, is given by

2 $$ {\hat{I}}_j^2=\frac{1}{M}{\sum}_{m=1}^M{\hat{I}}_j^2\left({T}_m\right) $$

where *M* is the number of iteration. The relative influence (or contribution) of each variable is scaled so that the sum adds to 100, with higher numbers indicating a stronger influence on the response. A threshold of 10% was set below which a variable is considered to have no substantial contribution to the model [33]. Variables that contributed less than 10% in both study zones were EVI, DEM, maximum night land surface temperature, Bio19, Bio18, maximum day land surface temperature, LULC, mean and minimum night land surface temperature. In addition to the above variables, Bio17, distance to an inland water body, population density, and mean day Land surface temperature also had less than 10% contribution in the northern zone; whereas improved housing, Bio12, and minimum night land surface temperature had an insignificant contribution to the model for the southern zone.

### Model selection

Six model classes i.e., generalized linear models (GLM) [31], multivariate adaptive regression splines (MARS) [19], artificial neural networks (ANN) [21], generalized boosted models (GBM) [15], Random Forests (RF) [4], and surface range envelope (SRE) [3] were tested using Biomod2 package in R [36]. Out of these, the Random Forest and GBM were the best performing models for this data and were therefore used for modelling and predicting LF suitable environments. Hundred (100) model runs for each algorithm was performed iteratively, and the evaluation values of each run were stored and then averaged to make the final result more robust. Model evaluation was performed based on the area under the receiver operating characteristic (ROC) curve. This measures the ability of the final ensemble model to fit the presence-absence data and predict across unsampled locations.

## Results

### Distribution of *mf* in Ghana

The distribution of *mf* cases from 430 communities surveyed in Ghana showed that LF infection was mainly found in the northern, southern and some parts of Ghana’s middle belt. The presence points indicated in red in Fig. 2 showed *mf* occurred along coastal communities in southern Ghana (i.e., Western and Central Regions). In the northern sector, *mf* cases were widespread in most of the districts, which also had a very high incidence compared to southern Ghana.

### Model performance

Model performances of six species distribution algorithms for the CW, NZ, and SZ models are shown in Table 2. Judging by AUC, sensitivity (percentage of presence correctly predicted), and specificity (percentage of absence correctly predicted) values, RF and GBM models outperformed the ANN, SRE, MARS, and GLM. AUC values between 0.5–0.6 indicate a failed model performance, whereas 0.6–0.7 represent poor model quality; 0.7–0.8 represent models with fair performance and 0.8–0.9, indicate a good model performance [24]. Overall, the RF was of the best quality for the CW model as well as the NZ and SZ models (Table 2). For further evaluation, results of the model with AUC ≥ 0.8 only (i.e., RF and GBM) were considered for the ensemble modelling.

**Table 2:** Calculated AUC, sensitivity and specificity values of different SDM algorithms for mf occurrence in CW, NZ and SZ

Model	AUC CW	Sensitivity CW	Specificity CW	AUC NZ	Sensitivity NZ	Specificity NZ	AUC SZ	Sensitivity SZ	Specificity SZ
**GBM**	0.95	92.23	86.91	0.94	91.00	91.00	0.91	86.5	88.03
**RF**	0.97	94.99	93.64	0.98	95.51	95.55	0.95	92.5	97.04
**GLM**	0.83	88.55	71.55	0.84	79.63	82.81	0.82	72.4	87.35
**ANN**	0.82	84.61	73.27	0.71	86.38	52.16	0.79	64.95	89.36
**SRE**	0.67	76.57	58.12	0.64	7.74	51.2	0.44	97	3.08
**MARS**	0.87	84.73	80.82	0.82	80.22	77.72	0.77	61.8	91.73

### Influence and importance of risk variable in northern and southern Ghana

Variable importance was evaluated for environmental, socioeconomic, and climatic variables, as shown in Table 3. Here, it was observed that in all the two zones and also in comparison with the countrywide analysis, distance to stable night light was an important variable although a weak one for both GBM and RF algorithms: CW (0.10 and 0.14, respectively), NZ (< 0.01 and 0.17, respectively) and SZ (0.03 and 0.13, respectively). Besides, weak but important values were computed for variables such as terrain slope for NZ (0.10 and 0.18) and SZ (0.04 and 0.10) and improved housing for CW (0.09 and 0.10) and NZ (0.13 and 0.16) for both algorithms, respectively. On the other hand, while Bio 12 was of the highest importance for both algorithms in CW (0.43 and 0.31) and NZ (0.63 and 0.36, respectively), proximity to water bodies was given the highest importance for the same algorithms in SZ (i.e., 0.59 and 0.41, respectively). Finally, the following variables of varying importance i.e., fair to weak values, were unique for the three zones: CW (maximum night land surface temperature and DEM), NZ (Minimum day land surface temperature), and SZ (Bio 17, Population density and Minimum day land surface temperature).

**Table 3:** Variables used in SDMs and difference in variable importance for the CW, NZ and SZ. The highest variable importance value for each model is highlighted with bold and underlined numbers

Variables	GBM CW	RF CW	GBM NZ	RF NZ	GBM SZ	RF SZ
bio17	–	–	–	–	0.04	0.10
bio12	**0.43**	**0.31**	**0.63**	**0.36**	–	–
NTL	0.10	0.14	< 0.01	0.17	0.03	0.13
Population	–	–	–	–	0.25	0.13
Slope	–	–	0.10	0.18	0.04	0.10
DEM	0.21	0.17	–	–	–	–
Proximity to Waterbodies	0.10	0.19	–	–	**0.59**	**0.41**
Improved Housing	0.09	0.10	0.13	0.16	–	–
Mean Day Land Surface Temperature	–	–	–	–	0.04	0.13
Minimum Day Land Surface Temperature	–	–	0.11	0.13	–	–
Maximum Night Land Surface Temperature	0.06	0.06	–	–	–	–

### Partial dependence plots of factors associated with *mf* transmission

**Fig. 3:**
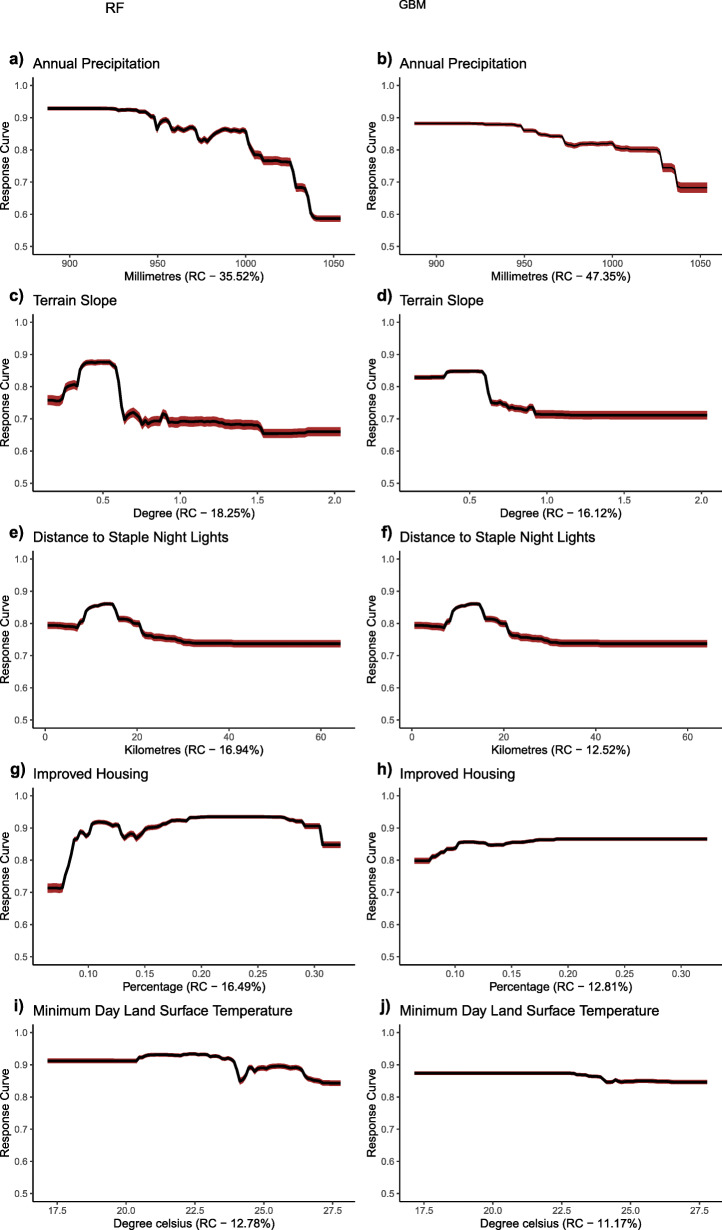
Response Curves of retained variables in the northern zone (NZ). The graphs were created for the RF and GBM species distribution models. (Plots were created by authors with RStudio Version 3.5.3 and no permissions are required to publish it)

**Fig. 4:**
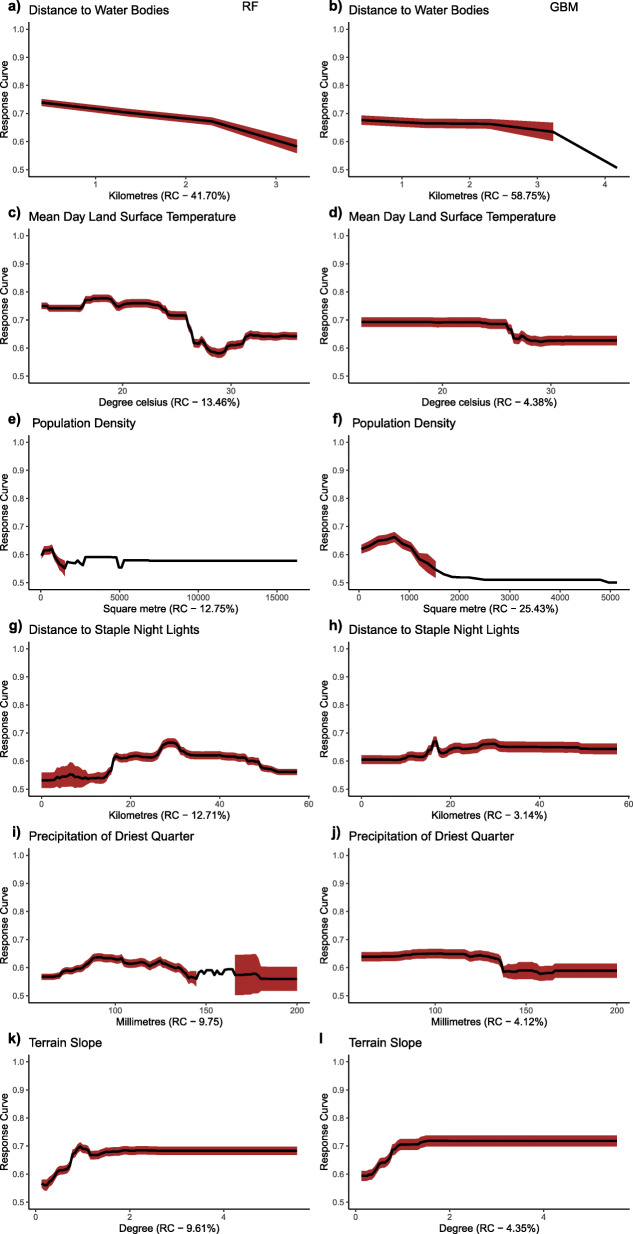
Response Curves of retained variables in the southern zone (SZ). The graphs were created for the RF and GBM species distribution models. (Plots were created by authors with RStudio Version 3.5.3 and no permissions are required to publish it)

Figures 3 and 4 show the response plot for each covariate for the RF and GBM models run with data from NZ and SZ, respectively. In northern Ghana, high suitability for *mf* was negatively associated with annual precipitation (i.e., rainfall values greater than 1000 mm result in a decrease in *mf* occurrence), high terrain slope, longer distance to the stable night light and minimum day land surface temperature values above 23 °C. There was a general increase of *mf* in areas with less housing improvement in northern Ghana and appeared to decrease in areas with greater than 30% improved housing (Fig. 3g).

**Fig. 5:**
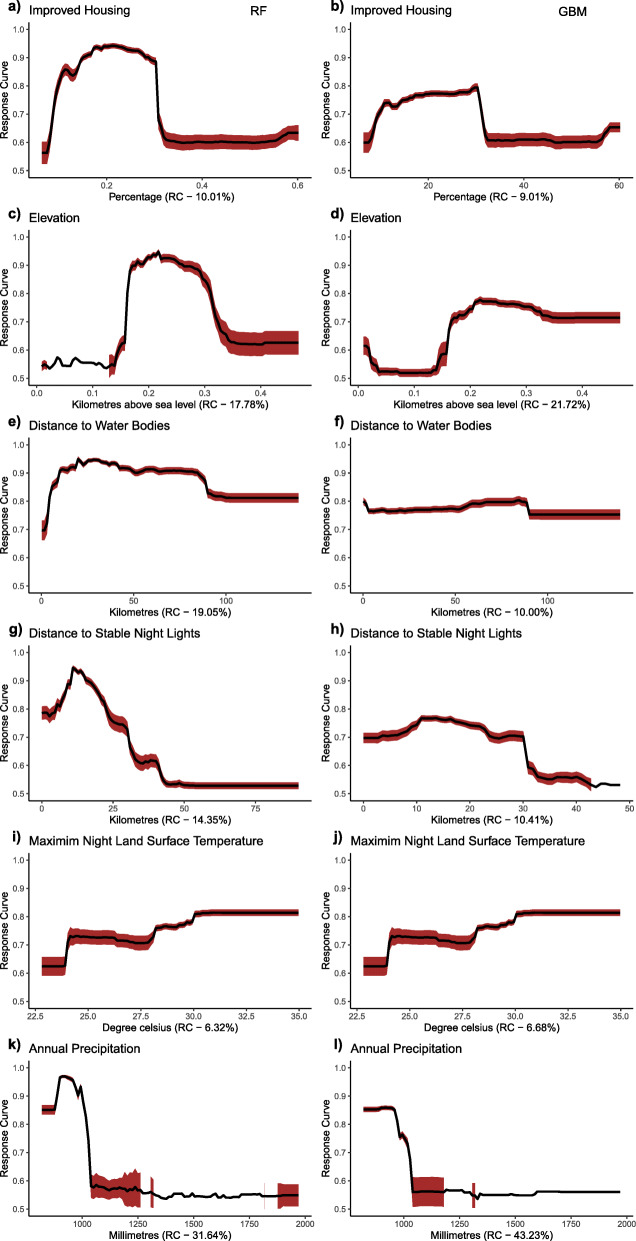
Response Curves of retained variables for the countrywide model (CW). The graphs were created for the RF and GBM species distribution models. (Plots were created by authors with RStudio Version 3.5.3 and no permissions are required to publish it)

High suitability values were associated with distance to water bodies and low suitability values associated with terrain slope in the south. It was observed that proximity to water bodies, population density, mean day land surface temperature showed a negative correlation with *mf* occurrence. In contrast, the increase in terrain slope and increased distance to stable night light showed a positive correlation with *mf* occurrence, as shown in Fig. 4. Response plot for CW (Fig. 5) showed consistency in the occurrence of *mf* with some similar covariates observed in the NZ and SZ, such as precipitation and distance to stable night light. The effect of the type of housing is better observed in the CW zone as *mf* occurrence decreased in areas with higher improvement in housing.

### Probability maps of LF occurrence

**Fig. 6:**
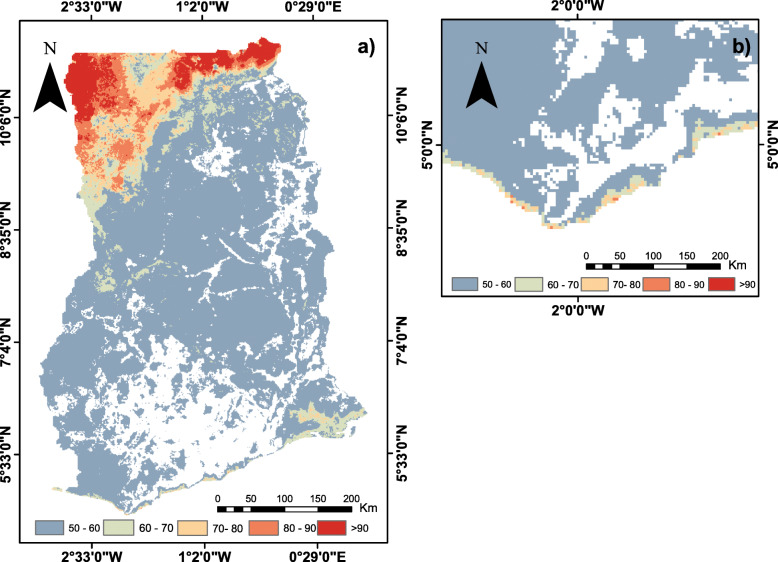
Probability maps of LF occurrence (%) generated with ensemble species distribution models, **a**) CW dataset and **b**) coastline exaggerated for cartographic purposes. Only the areas with a probability ≥0.5 are presented. Probabilities of ≥0.8 are highlighted in red shades and represent most likely transmission areas. (This map was generated by authors with ArcGIS V.10.6 software (ESRI, Redlands, CA, USA) and no permissions are required to publish it)

**Fig. 7:**
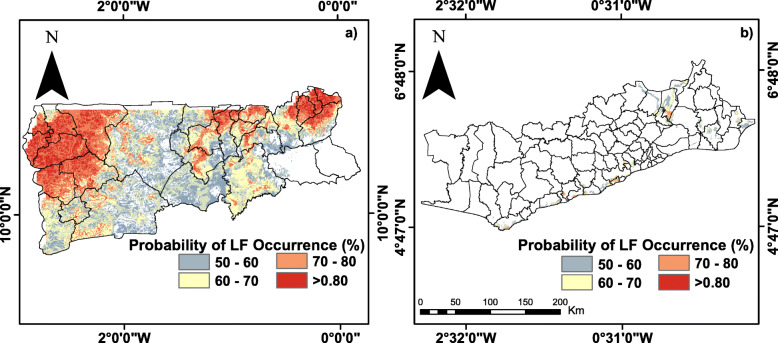
Probability maps of LF occurrence generated with ensemble species distribution models a) NZ and b) SZ. Only the areas with a probability ≥0.5 are presented. Probabilities of ≥0.8 are highlighted in red shades and represent most likely transmission areas. (This map was generated by authors with ArcGIS V.10.6 software (ESRI, Redlands, CA, USA) and no permissions are required to publish it)

The map for the CW zone shown in Fig. 6a represents discrimination of suitable and non- suitable environment for LF transmission over Ghana. Probability maps of CW, NZ, and SZ, shown in Figs. 6 and 7, highlight areas with zero or little occurrence probability (< 0.5) in large forest regions of Ghana. The maps as shown in Figs. 6a and 7a suggest that a larger portion of northwestern Ghana is environmentally suitable and better able to drive *mf* transmission. Areas of LF occurence rather shrinks sharply towards the northeastern part of Ghana showing high suitability in the extreme northern part of the country. Figure 6b exaggerates the coastline for cartographic purposes and suitable areas mirrored the SZ output as shown in Fig. 7b. These areas mainly correspond to mangrove ecosystems and freshwater swamps in the southern parts of the country.

## Discussion

Despite several years of mass drug administration and vector control measures against human lymphatic filariasis in Ghana, some areas continue to serve as hotspots for its transmission. LF occurrence in Ghana appears to vary from one location to another. In Ghana, two major endemic zones are known for LF, i.e., northern and southern zones. The mid-section has only a few cases of *mf* believed to have been imported from the north [23]. Understanding the differences in risk factors, i.e. environmental, climatic and socioeconomic covariates that drive *mf* transmission in the two study zones, is key to providing appropriate elimination strategies.

In this study, the evaluation of model performances revealed that RF and GBM algorithms performed better for all three zonal *mf* datasets. The two models in the current study showed that the AUC of success rate ranged from 0.95 to 0.98 and 0.91 to 0.95 for RF and GBM, respectively. This may be attributed to the fact that RF and GBM are better able to handle large covariates [37], as provided in this study.

The probability of *mf* occurrence is influenced by different combinations of variables in northern and southern Ghana. In the north, the occurrence of *mf* was influenced by low values of annual precipitation but decreased with high values above 1000 mm. The precipitation variable behaved differently in southern Ghana, with precipitation of the driest quarter sustaining LF transmission. In Ghana, heavy rainfall from April to June usually results in flooding in the northern region [27]. In the south, high rainfall patterns and low elevation, particularly along the coast, may result in surface water run-offs. These occurrences may sweep away breeding habitats reducing the survival of LF vector and subsequent transmission. However, rain availability especially in the coastal areas during the driest period of the year from late December to March, can create pockets of stagnant water bodies to sustain mosquito breeding, therefore increasing LF transmission. This implies that, whereas rainfall is needed for vector breeding, excessive rainfall could potentially result in flooding and sweeping away breeding sites [12]. Findings from this study are consistent with previous studies by Abiodun et al. [1], Cano et al. [6] and Eneanya et al. [17].

Similarly, the occurrence of *mf* declined with high land surface temperature during the day. This is consistent with adult mosquito survival and larval development, which suggests that both adult and larvae are unable to survive at high temperatures [25]. The response curve shows that minimum day land surface temperature values between 23 °C to 24.5 °C (GBM and RF, respectively) may increase the mortality rate of either larvae or adult mosquitoes in the north. Comparatively, vector survival is supported in the south beyond these temperature ranges until temperatures between 27 °C to 29 °C. This may result from thick vegetation cover or tree canopy in southern Ghana, which may create suitable conditions likely to sustain vector survival even at high temperatures. It was also observed that the probability of *mf* occurrence decreased with increasing terrain slope in both areas. This finding confirms with studies by Eneanya et al. [17]. What accounts for such observation is that steeper surfaces could lead to faster surface water run-off, thus decreasing water collection in pockets and eventually reducing breeding sites for vectors associated with LF transmission. In addition to poverty, areas of poor housing support transmission in the north.

Distance to stable night light was an important covariate for both northern and southern Ghana as well as at the countrywide scale. The response curve in the north shows that suitable areas for *mf* occurrence were generally rural and poor communities. In the south, some communities located in peri-urban to urban communities had a high probability of *mf* occurrence. This is true because some coastal communities located in peri-urban areas in the Western region have high *mf* prevalence.

Although some areas of the two the study zones (north and south) were suitable for *mf* occurrence, there exists slight differences in the suite of risk factors. This implies that any effort or strategy intended to eliminate the disease should consider unique conditions prevailing at a relatively fine spatial scale. The major limitation of the study was that the models did not consider important demographic risk factors at the community or individual level that are likely to improve the predictions. Finally, a larger sample size could lead to more precise predictions.

## Conclusion

This study has demonstrated that different variable combinations influence the occurrence of lymphatic filariasis in northern and southern Ghana. For both zones, the disease is highly prevalent in poor rural communities in low lying areas. In northern Ghana, areas suitable for transmission are relatively warm, low lying rural communities with poor housing, especially those characterized by mud houses. Besides, mean annual precipitation between 900 mm to 1000 mm provides a conducive environment for LF transmission. Similarly, rural, poor, low-lying, and most coastal communities in the south present a suitable environment for LF transmission. However, some peri-urban areas along the coast were also observed to be suitable areas. Generally, the infection is efficiently transmitted in warm lowland communities within 2 km of inland water bodies such as mangroves, lagoons, and rivers in the south. Moreover, rainfall within the relatively warm part of the year was identified as an important risk factor as it may contribute to the formation of stagnant water bodies suitable for mosquito breeding. Interventions such as improvements in housing and sanitary conditions may reduce LF transmission in endemic areas. The findings of the present study can be utilized by policymakers in advancing evidence-based strategies to eliminate LF.

## Data Availability

The data sources are publicly available online and can be accessed from the following publications: ([2, 22].

## Abbreviations

GLM: Generalized linear model
RF: Random Forests
GBM: Generalized boosted model
ANN: Artificial neural network
BRT: Boosted regression trees
SRE: Surface range envelop
MARS: Multivariate adaptive regression splines
SDMs: Specie distribution models
EVI: Enhanced vegetation index
MODIS: Moderate-resolution imaging spectro-radiometer
DEM: Digital elevation model
LULC: Land use land cover
ETM: Enhanced thematic mapper
VIF: Variance inflation factor
CV: Cross-validation
USGS: United States geological service
CW: Countrywide
NZ: Northern Zone
SZ: Southern Zone
AUC: Area under the curve
ROC: Receiver operating characteristic curve
MDA: Mass drug administration
NTDs: Neglected tropical diseases
lr: Learning rate
tc: Tree complexity
nt: Number of trees
LF: Lymphatic Filariasis
mf: Microfilaria
